# Explore the practice and barriers of collaborative health policy and system research-priority setting exercise in Ethiopia

**DOI:** 10.1186/s12961-024-01151-5

**Published:** 2024-05-30

**Authors:** Binyam Tilahun, Getasew Amare, Berhanu Fikadie Endehabtu, Asmamaw Atnafu, Lemma Derseh, Kassu Ketema Gurmu, Endalkachew Delllie, Adane Nigusie

**Affiliations:** 1 Department of Health Informatics, Institute of Public Health, College of Medicine and Health Sciences, University of Gondar, Gondar, Ethiopia; 2 Department of Health Systems and Policy, Institute of Public Health, College of Medicine and Health Sciences, University of Gondar, Gondar, Ethiopia; 3 Department of Epidemiology and Biostatistics, Institute of Public Health, College of Medicine and Health Sciences, University of Gondar, Gondar, Ethiopia; 4 Universal Health Coverage Life Course Cluster, Health Systems Team, World Health Organization Country Office, Addis Ababa, Ethiopia; 5 Department of Health Policy and Management, Jimma University, Jimma, Ethiopia; 6 Department of Health Policy and Global Health, Addis Continental Institute of Public Health, Addis Ababa, Ethiopia; 7 Department of Health Promotion and Health Behaviour, Institute of Public Health, College of Medicine and Health Sciences, University of Gondar, 196 Gondar, Ethiopia; 8 eHealthLab Ethiopia, University of Gondar, Gondar, Ethiopia

**Keywords:** Collaborative health policy, Researchers’ priority setting, HPSR, Ethiopia

## Abstract

**Introduction:**

Collaboration is gaining prominence in the priority setting of Health Policy And System Research (HPSR). However, its practice and challenges are not well explored in Ethiopia. Understanding the practice and barriers of collaborative Health Policy and System Research will help design approaches and platforms for setting inclusive and participatory policy and system-level health research topics. This paper explores the practice and barriers of collaborative HPSR-priority setting exercise in Ethiopia.

**Methods:**

This study investigates the practice and barriers of collaborative health policy and system research priority-setting exercises in Ethiopia. Utilizing a mixed-methods approach, we conducted Key Informant Interviews (KIIs) and an online self-administered survey with open-ended questionnaires to capture diverse perspectives from stakeholders involved in the research priority-setting process. Through conventional content analysis, we identified key contents related to current practices, challenges, and opportunities for enhancing collaboration in health policy and system research prioritization.

**Results:**

Our findings reveal a complex landscape characterized by varying levels of stakeholder engagement, institutional capacity constraints, and competing priorities within the health research ecosystem. Despite notable efforts to foster collaboration, stakeholders identified persistent challenges such as limited resources, institutional fragmentation, and inadequate coordination mechanisms as barriers to effective priority-setting processes. The implications of our research extend beyond academic discourse, with direct relevance to health policy and system research practice in Ethiopia. By shedding light on the dynamics of collaborative priority-setting exercises, our findings offer valuable insights for policymakers, researchers, and practitioners seeking to enhance the effectiveness and inclusivity of health research prioritization processes. Addressing the identified barriers and leveraging existing strengths in the research ecosystem can contribute to more evidence-informed health policies and programs, ultimately improving health outcomes for Ethiopian populations.

**Conclusions:**

Most institutions do not apply health policy and system research-priority setting to conduct informed decision-making. The barriers explored were weak integration, lack of knowledge, system, and platforms for the priority setting of Health Policy and System Resreach. So, it is recommended to build skills of different actors in the Health Policy and System Research-priority setting exercise and design a system and platform to integrate different stakeholders for collaborative research topics priority setting.

**Supplementary Information:**

The online version contains supplementary material available at 10.1186/s12961-024-01151-5.

## Introduction

Collaboration in Health Policy and System Research (HPSR) and priority setting is gaining prominence, with funders, particularly those of applied health research, and research institutions increasingly expecting researchers to engage the wider communities and other stakeholders throughout the research process [1–4]. This involves concerned stakeholders when shaping research projects' design, conduct, and dissemination, setting their research topics/priority settings, and formulating their research questions [5].

Such commitment is seen as a key way to ensure that research projects ask the 'right' questions those that are responsive to demanding stakeholder identified needs and create 'better' knowledge that draws on and reflects a diversity of knowledge systems and is more widely shared beyond peer-reviewed journals and academic conference [6, 7]. HPSR looks for how different organizations and actors collaborate for the achievement of collective goals in the implementation of policy [8–11]. It has the prospective to reimburse for or resolve existing differences in power, privilege, and positionality and allow for sidelined voices and experiences to be represented in the production of scientific knowledge [12].

Yet, concerned bodies rarely collaborate in the HPSR-priority settings or conduct health research projects that aim to help them. To achieve greater inclusion for stakeholders in research, including during priority-setting, researchers must partner with different community-based organizations [8, 10, 13, 14].

The research on collaborative health policy and system research priority-setting in Ethiopia offers insights into overcoming barriers and fostering effective collaboration among stakeholders. Its findings can inform evidence-based decision-making, enhance policy implementation, and promote equitable health outcomes, guiding future practices for policymakers, programmers, and researchers [15, 16].

Accordingly, many of the universities in Ethiopia now intend to develop collaboration strategies that call for undertaking research in partnership with community-based organizations. Community-based organizations have strong networks with their communities, including marginalized ones, developed through grassroots work and outreach activities.

However, in most Ethiopian universities, the collaboration among concerned bodies for the HPSR-priority setting exercise is at an infant stage. There are also many ups and downs in implementing partnerships for the HPSR-priority settings.

This study aims to explore the practice and barriers of collaborative health policy and system research priority-setting exercises in Ethiopia. The specific objectives guiding our research are as follows:

1. To examine current practices and processes of collaborative research priority-setting in the Ethiopian health context.
2. To identify the main barriers and challenges encountered by stakeholders involved in collaborative research prioritization efforts.
3. To explore opportunities for enhancing collaboration and improving the effectiveness of research priority-setting processes in Ethiopia.

By addressing these objectives, we seek to gain a comprehensive understanding of the dynamics shaping collaborative health policy and system research prioritization in Ethiopia. Through qualitative interviews and survey data analysis, we aim to generate insights that can inform policy and practice and contribute to strengthening the evidence-to-policy interface in the Ethiopian health system.

## Methods

### Research team and reflexivity

The key informant interview were conducted by the investigators (AN), (GA), and (ED) from the Institute of Public Health. The investigators (AN), (GA), and (ED) had extensive experience in qualitative research data collection and analysis. The credentials of the invsestigaotors AN, BT, AA, LD, and KKG were PhD; GA, BFE and ED were Maser of Public Health (MPH). All investigators are male and their occupation are researcher and lecturer at the time of this study. Prior to the study commencement the investigators were tried to estabilesh a relationship with the study participants. In terms of reflexivity, the investigators disclosed their identities to the participants and provided an overview of the study's objectives, as well as the rationale behind conducting the research.

### Study design

A descriptive qualitative study design was employed from January 2023 to July 2023. our decision to utilize the descriptive qualitative study design is underpinned by our unwavering commitment to methodological rigor and the accessibility of our research approach. Despite our considerable experience in qualitative research, we recognize the unique strengths of this approach in facilitating a comprehensive exploration of the practice and barriers of collaborative health policy and system research priority-setting exercise in Ethiopia. Through rigorous adherence to qualitative research principles and a dedication to making our research findings accessible to diverse stakeholders, we aim to generate insights that contribute to meaningful advancements in health policy and system research both locally and globally. In-addtion the descriptive qualitative study design capacity to provide a holistic understanding of the complex phenomenon under investigation, its contextual sensitivity to the Ethiopian context, its flexibility and adaptability to capture dynamic processes, and its accessibility to diverse stakeholders is some of the driving force to be coosen for our study. By leveraging the strengths of this methodology, we aim to generate valuable insights that can inform the advancement of collaborative health policy and system research priority-setting practices in Ethiopia and beyond.

## Theoretical framework

### Methodological orientation and Theory

Descriptive qualitative study is especially amenable to health environments research because it provides factual responses to questions about how people feel about a particular space, what reasons they have for using features of the space, who is using particular services or functions of space, and the factors that facilitate or hinder use [17]. Descriptive qualitative study suggests researchers tend not to penetrate their data in any interpretive depth. These studies present comprehensive summaries of a phenomenon or events. Qualitative descriptive designs tend to be eclectic methodologically and are based on the general premises of constructivist inquiry. The qualitative description is an excellent methodological choice for the healthcare environment designer, practitioner, or health sciences researcher because it provides rich descriptive content from the subjects' perspective [17].

### Participant selection/description of sample

The study group comprised key informants from different levels: Policy personnel, Program personnel, research institutes[18], Universities, and Non-governmental organizations. The Health Policy and System Research institutes were also selected for the open-ended online self-administered survey.

### Rationale for online self-administered survey with open-ended questionnaire

We opted for an online self-administered survey using an open-ended questionnaire as a complementary data collection method alongside Key Informant Interviews (KIIs). This decision was made for several reasons:

#### Accessibility and reach

An online survey allows us to reach a wider audience of stakeholders beyond those who may be accessible for face-to-face interviews. This method enables us to capture diverse perspectives, thereby enhancing the richness and depth of our data.

#### Convenience and flexibility

Online surveys offer respondents the convenience of participating at a time and place of their choosing, thus minimizing logistical barriers to participation. This flexibility is particularly advantageous in a context like Ethiopia, where stakeholders may have competing priorities and limited availability for in-person interviews.

#### Anonymity and candid responses

The self-administered nature of the online survey encourages respondents to provide candid and honest feedback, as they may feel more comfortable expressing their opinions anonymously. This can lead to more open and reflective responses, enriching the qualitative data collected through the survey.

### Qualitative nature of data

While online surveys are often associated with quantitative data collection, the use of open-ended questions in our questionnaire transforms this method into a qualitative data collection tool. Open-ended questions allow respondents to provide detailed, narrative responses, offering insights into their perspectives, experiences, and attitudes related to the research topic. Therefore, despite the online platform, the data collected through the open-ended questionnaire are qualitative in nature, aligning with the exploratory aims of our study.

We also acknowledge the challenge of obtaining adequate responses to open-ended questions via an online platform. To mitigate this challenge, several strategies were employed:

#### Clear and concise question design

The open-ended questions were carefully crafted to be clear, concise, and relevant to the research objectives. This increased the likelihood of respondents understanding the prompts and providing substantive responses.

#### User-friendly interface

The online survey platform utilized a user-friendly interface, making it easy for respondents to navigate and complete the questionnaire. Clear instructions and prompts were provided to guide respondents through the survey process.

Regarding the level of engagement and response Quality, respondents demonstrated a satisfactory level of engagement with the questionnaire, as evidenced by the completion rates and the quality of responses received. Although certain participants may have offered concise or superficial responses to open-ended inquiries, the majority offered detailed and insightful responses, indicating a meaningful level of engagement with the research topic.

### Sampling

Intensity purposive sampling was used to recruit study participants for key-informant interviews (KIIs) and online self-administer surveys. Intensity purposive sampling aims to develop a comprehensive understanding of the current practice and barriers of collaborative HPSR- priority setting exercisers. Therefore, the KII participants were selected based on their position and engagement in the decision-making, evidence-generation, synthesis, and research practices. Besides, those with information-rich cases and first-hand knowledge about the HPSR-priority setting practice were considered. These stakeholders, as influential actors, with their particular expertise and understanding, can provide insight into the nature of problems and recommend solutions [18].

A list of potential participants was determined, considering their close linkage with the health system research and their knowledge of the phenomenon of interest. In creating this list, we tried to get diverse representatives from different backgrounds, groups, or segments. This diversity of Key informants provides a broad range of perspectives. If we only interview people of a particular background or segment, we may end up with one-sided or biased results. Interviewing key informants from various segments allows us to look at varying perspectives and underlying issues or problems.

### Rationale for participant selection

1. Policymakers**:** Policymakers play a central role in shaping health policy and system research agendas and are key stakeholders in research priority-setting processes. Their perspectives and experiences provide critical insights into the interface between research and policy, highlighting opportunities and challenges for collaboration.
2. Policy experts**:** Experts with specialized knowledge in health policy and system research bring a depth of understanding and expertise to discussions around research prioritization. Their contributions enrich the dialogue on research priorities and inform evidence-based policymaking.
3. Academics and researchers**:** Academics and researchers contribute to the generation of new knowledge and evidence through their research endeavors. Their perspectives on research priorities, methodologies, and dissemination strategies are essential for informing collaborative priority-setting processes and ensuring the relevance and rigor of health policy and system research.

### Participant description

Participants in this study were selected based on their roles, expertise, and involvement in health policy and system research in Ethiopia. Demographic characteristics such as age, gender, and professional affiliation varied among participants, reflecting the diversity of perspectives within the stakeholder groups. Relevant background information, including academic qualifications, professional affiliations, and years of experience in health policy and system research, was collected to contextualize participants' perspectives and contributions to the study.

### Participant selection criteria

- Expertise: Participants were selected based on their expertise and experience in health policy and system research, ensuring that they could provide informed insights and perspectives on the research prioritization process.
- Involvement: Preference was given to participants actively engaged in health policy and system research activities or research priority-setting initiatives in Ethiopia, as their firsthand experiences and observations were deemed particularly valuable for the study.
- Diversity: Efforts were made to ensure diversity in participant selection, including representation from different institutions, geographic regions, and professional backgrounds, to capture a wide range of perspectives and experiences related to collaborative research prioritization efforts.

By purposefully selecting participants from these stakeholder groups, we aimed to capture a comprehensive range of perspectives and insights relevant to the collaborative health policy and system research priority-setting landscape in Ethiopia.

### Method of approach

The study participants were approached using a face-to-face interview in a safe place and online self-administered surveys using an open-ended questionnaire from the selected organazations.

### Sample size

The number of participants interviewed was determined by information saturation, which means that the information generated from repeated interviews becomes saturated [19, 20]. Thus, a purposively selected twelve key informants and six online self-administered surveys using an open-ended questionnaire were done. Once recruited and agreed to participate, consent was taken for the interview.

### Key informants

For the selection of key informants, a purposive sampling approach was employed to identify individuals with relevant expertise and experience in health policy and system research in Ethiopia. Key informants were recruited based on their roles, expertise, and involvement in research prioritization processes. Efforts were made to ensure diversity in participant selection, including representation from policymakers, policy experts, academics, and researchers across different institutions and geographic regions. Recruitment criteria also prioritized individuals actively engaged in health policy and system research activities or research priority-setting initiatives.

### Online survey participants

Participants for the online surveys were recruited through various channels, including professional networks, research institutions, and relevant organizations involved in health policy and system research in Ethiopia. The recruitment process aimed to reach a diverse range of stakeholders interested in or involved in research priority-setting processes. Participants were invited to participate in the online survey via email or through announcements on relevant platforms or mailing lists. The invitation included information about the purpose of the study, eligibility criteria, and instructions for accessing and completing the survey.

### Saturation in qualitative data collection

Saturation, or the point at which no new themes or insights emerge from additional data collection, is an important consideration in qualitative studies. Saturation was assessed iteratively during the data collection and analysis process by reviewing and comparing emerging themes and insights across interviews and survey responses. Data collection continued until thematic saturation was achieved, indicating that no new information or perspectives were emerging from additional interviews or survey responses. This iterative approach ensured the adequacy and comprehensiveness of the qualitative data collected for our study.

### Non-participation

No individuals refused in this study.

### Setting

#### The setting of the data collection

The data were collected in a quiet, secure, and comfortable place with minimum sound disturbance and voice to maintain the quality of the recording and facilitate open discussion. Interviewees determined the time and place of the interview.

#### The setting of the study area

The study was conducted at different levels of the Ethiopian health system that can influence the health policy issue, including Universities, research institutes (from regional and national levels),

Federal Ministry of Health (FMoH), Regional Health Bureaus (RHBs), and developmental partners.

#### Presence of non-participants

There are no other participants in the study except the participants and researchers.

### Data collection

#### Interview guide

We used a pilot-tested semi-structured interview guide prepared in English and translated into Amharic to elicit data details through probes. The interview guide for the KIIs and online self-administered survey questinnaire was developed separately based on literature related to the main research questions. The interview guides included six broad questions with suggested probes for the KIIs (Additional file 1) and the self-administered questions (Additional file 2) separetly. The guide was developed to capture participants' views on the Practice and barriers to HPSR-priority setting exercise in Ethiopia.

The interview guide was developed based on the study objectives and relevant literature on collaborative health policy and system research priority-setting. It included open-ended questions designed to elicit detailed responses from participants about their experiences, perspectives, and challenges related to collaborative research prioritization in Ethiopia. Probing questions were used to explore emerging themes in greater depth.

#### Number of interviews conducted

A total of 12 Key Informant Interviews (KIIs) were conducted with participants representing various stakeholder groups involved in health policy and system research prioritization in Ethiopia. Efforts were made to achieve diversity in participant demographics and professional backgrounds to ensure a comprehensive range of perspectives.

The online survey guide included an online self-administered questionnaire with open-ended questions related to collaborative health policy and system research priority-setting. The questionnaire was designed to capture similar themes as the interview guide, allowing for triangulation of data from different sources.

#### Online survey response rate

The response rate for the online survey was 100%. Efforts were made to maximize survey participation through targeted recruitment strategies, and reminders for completion.

### Repeat interviews

Repeat interviews were not carried out.

### Audio recording

The research used audio recording to collect the data.

### Field notes

The investigators made field notes during and after the interview. We made field notes for documenting needed contextual information. We contributed rich descriptive detail about the context of statements made, supplementing the recorded and transcribed participant statements and infusing the record with more significant meaning. In addition, we made field notes to clarify who the speaker was when recorded voices sounded similar. And to describe changes in body language, long pauses, facial expressions, making or losing eye contact, or other events that can help interpret the meaning from the context of what is said.

### Duration of the interview

The duration of the interviews, on average, is one hour and 35 min.

### Data saturation

In the current study, the term data saturation refers to the point in data collection when new interviews produce little or no new information to address the research question. No new information shall exist to get a higher degree of saturation. Based on the existing literature, a minimum of 12 interviews is typically needed. We also applied the more conservative approaches of operationalizing saturation to be confident enough in our conclusion of reaching saturation [21].

### Transcripts returned

The transcripts were not returned to participants for comment and correction because the principal investigators had a prolonged engagement in the data collection process.

### Data analysis

Two well-experienced researchers performed the data analysis. The researchers had taken training on qualitative data analysis methods using software. They had experience in qualitative data analysis methods, teaching a course on qualitative research methods and analysis for Ph.D. and Master students, and facilitating and delivering qualitative data analysis methods training for public health experts, lecturers, and researchers. Conventional content analysis was utilized for the analysis of the data. The conventional content analysis describes a phenomenon where existing research and theory are limited. Data are collected from open-ended questions, read word for word, and then coded. Notes are made, and codes are categorized. Steps for the data analysis: (1) a coding manual containing a beginning list of codes derived from the theoretical framework and literature and preliminary data analysis was developed before initiating data collection. Codes are action-oriented words or labels assigned to designated portions (chunks or meaning units) of text reflecting themes or topics that occur with regularity. (2) Each transcribed document was formatted with wide right margins, allowing the investigator to apply codes and generate marginal remarks by hand. (3) The investigator took sentences or paragraphs in the transcripts and divided them into meaning units, segments of text containing a single idea. 4) Conceptually similar codes were organized into categories (coding groups of coded themes that were increasingly abstract) by revisiting the theory framing the study. (5) During this analysis phase, pattern codes were revised and redefined in the coding manual, and exemplars were used to clarify the understanding of each code. (6) Analytic memos: "brief or extended narrative that documents the researcher’s reflections and thinking processes about the data." Memos aided in data reduction by tying together different pieces of data into conceptual clusters. Memos were personal, methodological, or substantive. (7) Data displays (matrices) or visual representations containing concepts or variables helped analyze the data. (8) Finally, the data are represented in a creative but rigorous way that is judged to fit the findings best.

### Number of data coders

Two individuals have performed the coding independently after repeatedly reading the transcribed document.

### Description of the coding tree

All tape-recorded data interviews and field notes were transcribed verbatim to Amharic (the local language) after repeatedly listening to the records and then translated into English. The translated transcription documents were imported into Open Code 4.03 software for coding.

The analysis used the four theme development phases: 1. Familiarization with the data, 2. Revisit research objectives, 3. Develop a framework, and 4. Identify patterns and Connections. Central themes were constructed based on the natural meaning of the categories. The investigators cross-cheeked the themes that emerged after analysis with the respective quotes in each theme. The findings were reported by a detailed description and interpretation of the themes' meanings. Direct quotes from the participants were also included in the write-up of the findings to provide clear images for readers. The overall data analysis used an inductive approach, i.e., a data-driven coding process through the research team discussion was performed to identify themes. Finally, these study findings were reported based on the consolidated criteria for reporting qualitative research (COREQ) guidelines (Supplementary Document 1).

### Derivation of themes

The theme and sub-themes were derived from the data.

### Software used

Open code 4.03 software was used to manage the data.

### Participant checking

Member checking is a crucial component of qualitative research methodology, aimed at enhancing the credibility and trustworthiness of findings by soliciting feedback from participants on the accuracy and interpretation of the data. As a method of Member Checking, after completing the analysis of our data, we prepared a summary of the key findings and interpretations derived from the interviews and online survey responses. This summary was designed to succinctly capture the main themes, patterns, and insights that emerged from the data collection and analysis process.

To facilitate member checking, we utilized an online platform to share the summary of findings with participants. The online platform offered a secure and user-friendly environment for participants to access the summary document and provide feedback.

Participants were contacted via email or through the same online platform used for the initial data collection (in the case of online survey respondents). They were provided with a link or access instructions to view the summary document and invited to review it at their convenience.

In the invitation message, participants were encouraged to reflect on the accuracy, relevance, and completeness of the summary findings in relation to their own experiences and perspectives. They were invited to provide feedback on any discrepancies or misinterpretations they identified and to offer additional insights or clarifications as needed.

A specific timeframe was provided for participants to review the summary document and submit their feedback. This timeline was established to ensure timely collection of responses while allowing participants sufficient opportunity for thoughtful reflection.

Periodic reminders were sent to participants who had not yet submitted their feedback to encourage their participation. These reminders reiterated the importance of member checking in validating the findings of the study and ensuring their voices were accurately represented.

Once all feedback had been received, it was systematically reviewed and analyzed by the research team. Each comment or suggestion provided by participants was carefully considered in relation to the original data and analysis. Where discrepancies or areas of ambiguity were identified, further clarification or follow-up may have been sought from participants.

### Trustworthiness

The trustworthiness of a study was ensured if the findings were credible. Experts reviewed the guides to ensure the quality of the data, and a pilot test was done to ensure the flow of the questions and cultural sensitiveness. The investigators also used simple language and descriptions in conducting the interview. An audit trail was considered a crucial technique to ensure the accuracy and credibility of the results. There was prolonged engagement with and persistent observations of research subjects by the principal investigators. The external audit was also considered to confirm the accuracy of the findings and to ensure the findings are supported by the data collected.

### Steps taken to ensure data analysis rigor

#### Conventional content analysis framework

Conventional content analysis was employed to analyze the qualitative data collected from interviews and open-ended survey responses. This involved a systematic process of coding, categorizing, and interpreting data to identify recurring themes and patterns.

#### Inter-coder reliability

To ensure rigor and reliability in data analysis, multiple coders independently analyzed a subset of interview transcripts and survey responses. Inter-coder reliability checks were conducted to assess agreement in coding decisions and ensure consistency in data interpretation.

#### Peer debriefing and member checking

Regular team meetings were held to discuss emerging findings, refine analytical frameworks, and validate interpretations through peer debriefing. Additionally, member checking was conducted to validate the accuracy and relevance of findings with participants, enhancing the credibility and trustworthiness of the study.

#### Reflexivity and audit trail

Researchers maintained reflexive journals to document their reflections, biases, and assumptions throughout the data collection and analysis process. An audit trail was established to track decision-making processes and ensure transparency and accountability in data analysis.

#### Triangulation of data sources

Data triangulation was employed to compare and contrast findings from different data sources (interviews, online surveys) and participant perspectives. This helped to enhance the validity and reliability of study findings by corroborating themes and insights across multiple sources.

## Findings

### Background information of the study participants

A total of 12 key informants and six online self-administered assessment surveys were conducted from the selected organizations. The participants were selected from the Ministry of Health (MoH), Ethiopian Health Information System (EHIS), Ethiopian Public Health Institute (EPHI), Armauer Hansen Research Institute (AHRI), regional health bureaus (RHB), development partners, and universities. The participants ranged in age from 35 to 44 years old and possessed 14–20 years of professional experience. Additionally, all participants held educational qualifications beyond the master's level (Table 1).

**Table 1:** Socio-demographic characteristics of participating in key informant interview and online survey, 2023

Code	Sex	Age	Work experience	Educational level	Position	Remark
01	M	48	23	Msc	Director	Partner
02	M	44	19	PhD	Director	FMoH
03	M	49	26	Professor	Head	University
04	M	48	24	Professor	Technical assitant	FMoH
05	F	36	13	MPH	Vice director	FMoH
06	F	40	17	PhD	Researcher	AHRI
07	M	42	18	PhD	Researcher	University
08	M	39	17	MPH	Director	RHB
09	M	40	16	MPH	Manager	Partner
010	M	45	21	PhD	Research expert	FMoH
011	M	37	14	MD	Director	RHB
012	M	39	16	MD	Director	FMoH
013	M	36	14	MPH	Director	EPHI
014	F	41	19	PhD	Researcher	University
015	M	38	15	PhD	Director	EPHI
016	M	41	17	PhD	Director	FMoH
017	M	46	22	PhD	Researcher	AHRI
018	M	44	18	MPH	Director	EHIS

Key: Participants from 013 to 018 were the online survey respondents

Many of the study participants reported that the understanding of the HPSR concept in their institution/staff is at a medium level. They also reported limited availability of HPSR-related evidence to support their institution's evidence-based decision-making process. However, they mentioned that there is a practice of formative research for problem identification and set their annual and monthly plan. In the decision-making process, most participants have no prioritization criteria because of unplanned activities from the higher officials.

**Fig. 1:**
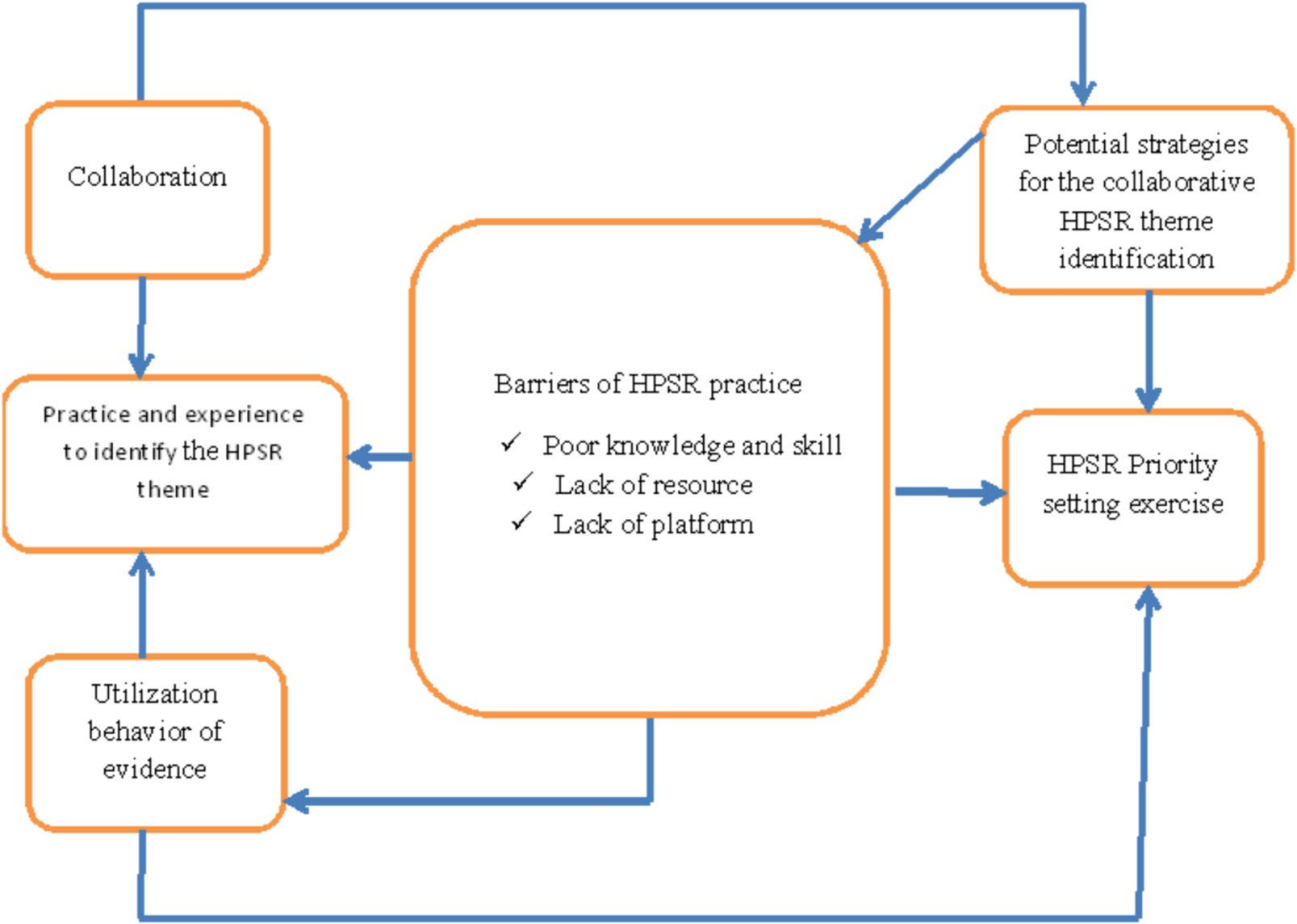
Visual conventional content analysis of the practice and barriers of collaborative health policy and system research-priority setting exercise in Ethiopia

Based on the data driven from this study, the primary focus of the study is categorized broadly into five key themes, namely: Practice and experience to identify the HPSR theme, Collaboration, Utilization behavior of evidence generated by the research, Barriers for the HPSR practice, and Potential strategies for the collaborative HPSR theme identification. In addition, each key theme is categorized into sub-themes (Fig. 1).

1. Practice and experience to identify the HPSR theme
  The main purpose of health policy and systems research (HPSR) is to inform and influence health policies and systems to pursue health goals. However, it was not well practiced in Ethiopia since it is an emerging field.
  Many study participants stated that there is no well-organized practice of HPSR theme identification in their respective organizations. The participants stated that they used the challenge faced to identify problem titles to survey to develop manuals and directives. This is supported by the following quote;
  "… *We are only searching the gaps and the problems we want to address, but we didn't map the problems and research agendas targeting the health system and policy-specific matters."*
  On the contrary, a few participants reported trying to set an agenda for HPSR even though it is not well practiced due to lack of evidence. This is supported by the following quote;
  *"Yes, my institution tries to set an agenda for HPSR since it is one of the thematic areas of our college…However, it is not well practiced. Most of the agendas are related to other public health topics. We have no adequate evidence for it."*
  Likewise, many participants reported that no structure or unit is assigned to facilitate the HPSR agenda-setting in their organization. This is supported by the following quote;
  "… *We don't have a unit specifically assigned for this role."*
  Those few participants stated that as they tried to practice the HPSR agenda setting, they did not have a unit/structure assigned. They report that the agenda-setting was done by senior researchers, the research assistance council (RAC), and volunteers from the academics and partners. This is supported by the following quote;
  *"Even though there is no established structure for this specific task, the program side mostly comes up with relevant research questions identified during program implementations. These questions are forwarded to be addressed by researchers, RAC members, or research institutions…."*
2. Collaboration practice
  Collaboration is vital in public health for the successful and sustained accomplishment of activities. These could strengthen the collaboration for problem identification, prioritization, implementation, and utilization of research findings.
  Many participants stated that there are poor collaborations between the research institutes, academics, partners, and policymakers. These could negatively affect the HPSR agenda settings. This is supported by the following quote;
  *"…As I have said before, there is poor collaboration. Everyone would go for individual activity; there is no collaboration trend. Sometimes, unfortunately, there might be collaboration, but it ceased when they accomplished that task…."*
  The health system searched for partners needing money other than the local research institutes. The researcher also goes for their personal development rather than finding out the real public health problems from the community. This is supported by the following quote;
  "… *The Practice of collaboration among universities, policymakers, and other key actors is limited to my understanding….The initiations are not guided by the system and structure but are based on individual efforts and interests. Besides, the practice related to health system and health policy related knowledge generation and collaborative agenda setting is also limited."*
3. Utilization behavior of evidence generated by the research
  The Practice of using the evidence generated by research is limited by most organizations, as stated by the participants. This is because no guiding principles emphasize the use of the generated evidence. Rather, the utilization of the evidence is based on individual efforts and interests. This is supported by the following quote;
  "… *Even if there are initiations here and there, it is below expectation. The initiations are not guided by the system and structure but are based on individual efforts and interests. Besides, the practice related to health system and health policy related knowledge generation and collaborative agenda setting is also limited."*
  No, a platform interconnects the researcher and others for proper use of the evidence generated. Those people from the health system were unfamiliar with what the researcher from the university did and vice versa. This is supported by the following quote;
  "… *We are trying to search the available evidence based on the needs of the users/ministry. However, there is no organized platform to access and map the research findings generated by the universities. Each university works independently, and there is no common platform to set the research agendas based on the real problems…."*
  The donor-driven activity mostly influenced the people from the health system. Therefore, rather than searching for evidence from the researcher, they (people from the health system) go to activities from the partners, which is very specific and doesn't consider the large public health importance. This is supported by the following quote;
  *"… Most of the time donor driven activity re-directs our plan. There is no alignment of activity. Most of the time, the donor activity was a specific area of interest and could be easily implemented. So, we need to implement it in other areas. The donors did not care about the government Issue."*
4. Barriers to the HPSR practice
  In the healthcare system of Ethiopia, there are many new issues and evolving roles for different stakeholders, as well as novel ways to study and influence health systems. The health of the population in Ethiopia is being challenged in different ways. The study participants listed a couple of barriers/challenges to practicing HPSR in Ethiopia's health. These challenges can be categorized as Poor Knowledge and skill on HPSR, Lack of resources, and lack of structure/ platform to link collaboration.

### Poor knowledge and skill on HPSR

The knowledge and skill of research enable one to focus on a specific goal, gather relevant information, and communicate findings to others. As HPSR is an emergent field, it lacks well-experienced professionals. Many participants stated that the lack of experienced researchers in HPSR requires them to focus only on formative research using survey methods. This is supported by the following quote;

"… *Most of the time burden of disease, service availability and coverage, basic operation researches is more dominant, and the attitude and need for the issue of HPSR is limited….one challenge is skill and knowledge-related gaps…."*

Similarly, they stated that there is a technical and conceptual gap about HPSR by most of the donors. The professionals from the partners and research institutes lack experience in implementing HPSR. This is supported by the following quote;

*"Since the field is new, technical and conceptual related gaps exist. The practice of HPSR-related evidence generation by universities and other research institutes is also limited…. Due to this, there are technical limitations in the health system and policy-related research topics."*

### Lack of resource

Resources are critical for any research activity to be successful. The HPSR needs a proper budget to run its effectiveness at the expected level. However, there is no such resource allocation for research activity in the Ethiopian health system.

Almost all the study participants stated that the lack of resources is a bottleneck for implementing the research activity, including the HPSR. They emphasized that no budget was assigned for the research activity. So that people are going for other partners who may have a budget for their activity. This is supported by the following quote;

*"… There are many challenges in the implementation of HPSR. The main challenge includes the low budget allocation for the research activity by the government.…"*

Likewise, with the lack of experienced experts in HPSR, few participants said there is poor utilization of skilled workforce. This is supported by the following quote;

*"… There is also poor utilization of the skilled manpower…."*

### No structure/platform to link collaboration

The platform is a strategy for developing the fundamental structure for an organization's overall efforts. Thus, structure/platforms can significantly accelerate learning by providing more real-time feedback loops, where system participants can see what results in their current actions produce, reflect adjustments, and refine their actions based on real-time feedback.

All participants of the current study reported that no platform/structure enables the integration of the research institute, academics, policymakers, and partners. There is a huge bridge between the academic institutes and the health system due to the lack of a platform that creates a collaborative wing between the two organizations. This is supported by the following quote;

*"… The other challenges are that the program leaders, the policymakers, and researchers from the university, college, and research institute have no communal platform for collaboration and coordination. Everybody was running separately; there was no common discussion in a comprehensive way to come up with a change.…"*

Similarly, academia was going only for personal development without considering the actual problems from the ground. The health system workers also run their activity without collaborating with the nearest research institute and academicians. The health system searches for partners other than the nearest universities due to the need for fund support. Even the universities themselves do not have any means of collaboration on the activity they perform; there is duplication of efforts. The policymakers also hesitate about the quality of evidence generated by the academicians, fearing improper utilization of the assigned funds for the activity. This is supported by the following quote;

*"…We didn't have a regular and structure-based collaboration platform. Besides, academic institutions are mostly interested in developing evidence targeting academic development and achievements. There are also hesitancies from the policymaker's side on the quality and policy relevance of the evidence generated by the academic institutions.…"*

## Potential strategies for the collaborative HPSR theme identification

The participants were given suggestions for potential strategies for the collaborative HPSR theme identifications, which should be emphasized. Among the suggested strategies;

- There should be a platform for the discussion, collaboration, and strengthening of program leaders, policymakers, and researchers.
- A central data warehouse/cloud should be established.
- There should be a standard policy brief preparation format that all universities could utilize.
- Conducting the capacity building in the area of HPSR.
- Resource and partner mapping in the area of HPSR.

"… *The structure and the system should be established. Policymakers should set agendas, share the research based on their needs and problems, and easily access the evidence generated. This system has to be established. …"*

## Discussion

This study explored the practice and barriers to health policy and system research-priority setting exercises in Ethiopia. Findings from this research are categorized into five themes: Practice and experience identifying the HPSR theme, collaboration, utilization behavior of evidence generated by the research, barriers to the HPSR practice, and potential strategies for the collaborative HPSR theme identification. Meanwhile, this study explored the current practices and barriers of collaborative health policy and system research-priority setting exercise in Ethiopia, which helps to fill the gaps in the collaboration process for the HPSR-priority setting exercise.

The health system encompasses all the organizations, institutions, and resources devoted to producing health actions that aim to improve health. So, the current findings could help to design strategies that could improve the collaboration in the HPSR-priority settings by proving feasible and useful strategy to fight against the barrier, explaining why the existing strategy is not successful, showing how it can be applied in Practice for the Ethiopian context, making ideas tangible; what its limitations are, and providing a new solution to a known problem and demonstrating the solution's efficacy [22–24].

Health research is a driving force for improving the performance of health systems and the health of individuals and populations [25–28]. However, the practice of HPSR theme identification was not well exercised in most organizations. Studies from Ethiopia and Ghana showed that policymakers and research institutes voice that the level of HPSR spending is inadequate [29]. This is because researchers in different disciplines often work in isolation, in a fragmented, competitive, and highly specialized activity. Furthermore, the overall emphasis of research priorities is greatly lop-sided by the bias of the funding organization [22, 30].

This study's active collaboration between health system researchers, decision-makers, and other research users was poor. The active collaboration could promote evidence-informed policy and policy-informed research [4, 31, 32]. Naturally, HPSR is inter-disciplinary, a blend of economics, sociology, anthropology, political science, public health, and epidemiology that together draw a comprehensive picture of how health systems respond and adapt to health policies and how health policies can shape and be shaped by—health systems and the broader determinants of health [4, 33]. Therefore, the concerned bodies must strongly commit to creating an active linkage and exchange between health system researchers, decision-makers, and other research users.

One of the fundamental principles of health systems research is that its production must balance its utilization. However, the current study revealed that the utilization behavior of evidence generated by research is limited and given less emphasis. In the developing world, including Ethiopia, there is a pushing and pulling of tasks between Policymakers, managers, and researchers. Policymakers and managers criticize that they frequently encounter research not relevant to real-life problems at the grassroots level, full of results expressed in obscure language, often published in unreachable journals. Conversely, researchers often criticize policymakers and health managers for ignoring research results, which are the fruit of careful work supported by substantial investment.

Meanwhile, funding agencies wonder how to demonstrate that investment in health systems research has made a difference [24, 34]. This implies that policymakers and managers need to increase research use in decision-making. In contrast, Researchers, research managers, and funding agencies need to pay more attention to understanding policy issues and facilitating research output.

The participants reported several critical barriers to the Practice of HPSR. Barriers to working together can be addressed by establishing linkages between institutions to foster the work of multidisciplinary teams capable of addressing issues in health policy and health systems [24, 35]. Poor knowledge and skills in HPSR are critical barriers to the Practice of HPSR-priority setting exercise. Linkages between institutions serve several functions, including capacity development through training, support for exchanging information, and collaboration between researchers [24, 36–38].

The participants suggested strategies for sustained collaborative HPSR theme identifications in the present study. Among the suggested strategies, the establishment of a platform for the discussion, collaboration, and strengthening of program leaders, policymakers, and researchers was commonly suggested by other scholars. This is because it is necessary to establish pragmatic definitions of 'health sector research' and 'non-health sector research', within a spectrum ranging from mainstream research that emanates from the health sector to research in non-health sectors that was never conceived as being health research but which could have relevance for health policy [39–42].

## Implications and recommendations

Our exploration of collaborative health policy and system research in Ethiopia has unveiled a multifaceted landscape characterized by diverse stakeholder engagement, institutional limitations, and competing priorities. Despite commendable efforts, persistent challenges such as resource constraints, institutional fragmentation, and inadequate coordination mechanisms hinder effective priority-setting processes. The implications of these findings extend beyond academic discourse, directly impacting health policy and system research practice in Ethiopia. To address these challenges and capitalize on existing strengths, we offer tailored recommendations for policymakers, researchers, and practitioners.

## Recommendations

I. Establish a Centralized Coordination Mechanism:
  - Create a centralized body tasked with coordinating health policy and system research activities, fostering collaboration among stakeholders, and harmonizing research priorities.
  - This mechanism should ensure representation from diverse stakeholders, including government agencies, academia, civil society organizations, and international partners, to promote inclusivity and accountability.
II. Strengthen Institutional Capacity:
  - Invest in building the capacity of research institutions and governmental agencies involved in health research to conduct priority-setting exercises effectively.
  - Provide training programs, technical support, and resources to enhance research skills, data management capabilities, and evidence synthesis expertise.
III. Foster multi-sectoral partnerships:
  - Encourage collaboration across sectors, including health, education, finance, and social welfare, to address complex health challenges comprehensively.
  - Facilitate partnerships between research institutions, policymakers, and implementers to ensure the translation of research findings into actionable policies and programs.
IV. Enhance resource mobilization:
  - Advocate for increased funding for health research, emphasizing its critical role in informing evidence-based policies and interventions.
  - Explore innovative financing mechanisms, such as public–private partnerships and research grants, to diversify funding sources and sustainably support research activities.
V. Promote knowledge translation and exchange:
  - Develop mechanisms for effective knowledge translation, dissemination, and utilization to bridge the gap between research evidence and policymaking.
  - Foster a culture of evidence-informed decision-making by promoting dialogue, workshops, policy briefs, and interactive platforms for sharing research findings.
VI. Prioritize equity and inclusivity:
  - Ensure that priority-setting processes are inclusive and transparent, incorporating the perspectives of marginalized populations, women, youth, and vulnerable groups.
  - Promote equity-focused research agendas that address disparities in health outcomes and access to healthcare services across regions and population groups.

## Strengths and limitations

An inclusive view of researchers, policymakers, managers, and partners on the Practice and barriers of HPSR-priority setting exercise was obtained from different stakeholder categories. This reflects a wide range of experience designing appropriate interventions for the barriers. As a limitation, the findings of this study can be applied to the Ethiopian health system structure only and cannot be taken to apply for another structure. Eventhough HPSR is interdisciplinary by nature, including economics, sociology, anthropology, political science, public health, and epidemiology, our team didn't consider inclusion of these important disciplines in participant selection.

## Conclusions

The current study has identified five themes. Namely, Practice and experience to identify the HPSR theme, Collaboration, Utilization behavior of evidence generated by the research, Barriers to the HPSR practice, and Potential strategies for collaborative HPSR theme identification. The Practice of collaborative HPSR-priority setting exercise in Ethiopia is at an infant stage. Therefore, it is recommended that: (1) Strengthen researchers' capacity to understand the national or subnational level policymaking process. This can be done by building relationships with relevant policy actors through in-person interactions, involvement in current policy discourse, identification and communication with policy entrepreneur(s), and serving as members of a task force, working group, or relevant national committee. (2) Policy-relevant evidence must be generated promptly to facilitate policy decisions. (3) Much more needs to be done, and the funding, research, and policymaking community must come together to facilitate the required practice of HPSR-priority setting exercise.

### Supplementary Information

Supplementary Material 1.

Supplementary Material 2.

Supplementary Material 3.

## Data Availability

Data will be available upon request from the corresponding author.

## Abbreviations

AHRI: Armauer Hansen Research Institute
APHI: Amhara Public Health Institute
COREQ: Consolidated Criteria for Reporting Qualitative Research
EHIS: Ethiopian Health Information System
EPHI: Ethiopian Public Health Institute
FMoH: Federal Ministry of Health
HPSR: Health Policy and System Research
KIIs: Key Informant Interviews
MoH: Ministry of Health
RHB: Regional Health Bureau
SDGs: Sustainable Development Goals
UHC: Universal Health Coverage
WHO: World Health Organization
