# Supplementary material for: Explore the practice and barriers of collaborative health policy and system research-priority setting exercise in Ethiopia
Source: Health Res Policy Syst. 2024 May 30;22:64. doi: 10.1186/s12961-024-01151-5 (PMC11138033; doi:10.1186/s12961-024-01151-5)
Supplement: Supplementary file 1 — Supplementary Material 1. [file 12961_2024_1151_MOESM1_ESM.docx]

**Title: Explore the Practice and Barriers of Collaborative Health Policy and System Research Priority-Setting Exercise in Ethiopia.**

**Key informant in-depth interview (KII) Guide for the selected key-informant participants**

General information

Name of Interviewer: _________________ Date: _________________________

Time Interview started: ________Time Interview ended: __________

Name of Region: ______________________ Name of Institution: ________________________

1. Demographic characteristics of participants in the interview

| No | Code of Participant | Sex | Age | Years of experience | Educational Level | Position |
| --- | --- | --- | --- | --- | --- | --- |
| 1 |  |  |  |  |  |  |

1. *How do you see the decision-making process like strategic plan development, action plans, and resource allocations in your organization? (Probe: Prioritization, or any other approach)*

*1.1. How priority settings activities were applied?*

*1.2. When priority settings activities were applied?*

*1.3. Why priority settings activities applied? (Probe: one management principle; efficiency, cost saving, timeliness, effectiveness)*

*1.4. Who is using the priority-setting principles? (Probe: All staff, low-level management, senior management?*

1. *How is the understanding of HPSR in your institution/staff?(Probing: any Capacity building,…)*
2. *How do you identify HPSR themes in your institution? (Probing: how it was conducted, who was participated, how was the procedure and what criteria were used?, If No experience of HPSR theme selection: (what was the reason, skill, system, collaboration, interest, plan, awareness)*
3. *What were the potential barriers or challenges for the HPSR practice in Ethiopia? (Probe: skill, collaboration, priority, demand?*
4. *How do you see your collaboration with the research/health system institutions? (Probe: Research/evidence generation, capacity building, knowledge sharing/exchange, community service, implementation science research; any challenges of collaboration)*
5. *Any opinion/suggestions you want to say regarding…….*
