# Supplementary material for: Explore the practice and barriers of collaborative health policy and system research-priority setting exercise in Ethiopia
Source: Health Res Policy Syst. 2024 May 30;22:64. doi: 10.1186/s12961-024-01151-5 (PMC11138033; doi:10.1186/s12961-024-01151-5)
Supplement: Supplementary file 2 — Supplementary Material 2. [file 12961_2024_1151_MOESM2_ESM.docx]

**To: ___________________**

**---------------------**

**Subject: - Invitation to fill the below questions for the HPSR priority setting exercise practice and experience**

As you know the University of Gondar in collaboration with Ethiopian Public Health Institute (EPHI) are planning to conduct the Ethiopia HPSR needs using the collaborative and participatory agenda setting. The HPSR agenda setting helps to have a common research area with researchers, academicians, policy makers and funder. Besides, the common agendas setting also helps to have a strategic resource mapping for the health policy and system evidence generation and efficient resource allocation. To conduct the project we are searching for the practice and experience of your institution in the HPSR agenda and priority setting exercise.

Therefore, you are kindly invited to reflect your institution practice and experience related to the HPSR agenda and priority setting practice for the listed questions.

We thank your contribution and time.

**Title: Explore the Practice and Barriers of Collaborative Health Policy and System Research Priority-Setting Exercise in Ethiopia.**

**Open guiding survey questions for the selected organazations**

1. *How is the understanding of HPSR concept in your institution/staff? (Did the staffs have better understanding or are there any limitations? Rate it*
   - 1. *Very good understanding*
     2. *Good understanding*
     3. *Medium*
     4. *Poor understanding*
     5. *Very poor understanding*
2. *How is the availability of HPSR related evidences to support your institution evidence based decision making process?*
3. *Enough evidence*
4. *Limited evidence*
5. *No evidence*
6. *How is the practice and experience of your institution in setting the HPSR agendas?*
   1. *Does the institution has the practice and culture of setting HPSR research agendas*

*Response___________________________________________________________________________________________________________________________________________________________________________________________________*

- 1. *If yes for question 1.1, how the HPSR agenda setting was done?(who are the participants and how was the procedure)*

*Response___________________________________________________________________________________________________________________________________________________________________________________________________________________________________________________________________________________________________________________________________________*

- 1. *Does the institution has conduct a priority setting exercise for the HPSR agendas?*

*Response___________________________________________________________________________________________________________________________________________________________________________________________________________________________________________________________________________________________________________________________________________*

- 1. *If yes for question 1.3, how the priority setting procedure or exercise was conducted? (the criteria used and the participants and the scoring procedure)*

*Response___________________________________________________________________________________________________________________________________________________________________________________________________________________________________________________________________________________________________________________________________________*

- 1. *Is there any structure or platform to share the research findings to the decision makers?*

*Response____________________________________________________________________________________________________________________________________________________________________________________________________*

- 1. *How it the practice of using the university research findings by different level of policy and decision makers?*

*Response____________________________________________________________________________________________________________________________________________________________________________________________________*

- 1. *What is main outcome of the researches conducted by the universities? (Policy and guideline development, academic development, resource mapping…?*

*Response____________________________________________________________________________________________________________________________________________________________________________________________________*

1. *How is the practice of setting common HPSR or other research agendas with the collaboration of policy makers, researchers, implementers and funders? (are there any platforms, structures or other approaches to set common research agendas)*

*Response_______________________________________________________________________________________________________________________________________________________________________________________________________________________________________________________________________________________________________________________________________________________________________________________________________________________________________________________________________________________________________________*

1. *Who is the practice and experience of the institution in conducting a collaborative research or evidence generation with research institutions and academicians? ((Probe: Research/evidence generation, capacity building, knowledge sharing/exchange, community service, implementation science research; any challenges of collaboration)*

*Response________________________________________________________________________________________________________________________________________________________________________________________________________________________________________________________________________________________________________________________________________________________________*

1. *What do you think about the challenges/barriers of participatory and common HPSR agenda setting?*

*Response________________________________________________________________________________________________________________________________________________________________________________________________________________________________________________________________________________________________________________________________________________________________*
